# Antiadhesive and antibiofilm potential of *Bacillus clausii* derived biosurfactant against *Streptococcus mutans*, *Enterococcus faecalis*, and *Candida albicans* biofilms

**DOI:** 10.1186/s12866-026-05270-7

**Published:** 2026-06-26

**Authors:** Zahra Khairunnisa, Wan Himratul Aznita Wan Harun, Isty Adhitya Purwasena, Dinda Kurnia Fitri, Nora Sakina Mohd Noor, Noor Hayati Azami, Arief Cahyanto, Myrna Nurlatifah Zakaria

**Affiliations:** 1 Faculty of Dentistry, Department of Restorative Dentistry, Universiti Malaya, Kuala Lumpur, 50603 Malaysia; 2 Faculty of Dentistry, Department of Oral Biology, Universitas Jenderal Achmad Yani, Cimahi, 40525 Indonesia; 3 Faculty of Dentistry, Department of Oral & Craniofacial Sciences, Universiti Malaya, Kuala Lumpur, 50603 Malaysia; 4 Department of Microbiology, School of Life Sciences and Technology, Institut Teknologi Bandung, Bandung, 40132 Indonesia; 5 College of Dentistry, Department of Clinical Sciences, Ajman University, P.O.Box: 346, Ajman, United Arab Emirates; 6 Centre of Medical and Bio-allied Health Sciences Research, Ajman University, P.O.Box: 346, Ajman, United Arab Emirates

**Keywords:** Antiadhesive, Antimicrobial, *Bacillus clausii*, Biofilms, Biosurfactants, *Candida albicans*, *Enterococcus faecalis*, Lipopeptides, Oral Health, *Streptococcus mutans*

## Abstract

**Background:**

Oral biofilms are principal drivers of oral and dental diseases such as caries, periodontal disease, endodontic infections, and candidiasis. Conventional biocides can reduce microbial load but may irritate tissues and disturb oral homeostasis, limiting prolonged use. Biosurfactants represent a sustainable alternative strategy by modulating adhesion and biofilm structure rather than relying solely on direct bactericidal activity. Lipopeptide biosurfactants are particularly attractive due to their amphiphilic structure, surface activity, and reported antibiofilm properties. F7 is a crude biosurfactant produced by *Bacillus clausii* isolated from a crude-oil reservoir in Sumatra, Indonesia. This study evaluated its physicochemical properties and antiadhesive/antibiofilm activity against three clinically relevant oral pathogens: *Streptococcus mutans*, *Enterococcus faecalis*, and *Candida albicans*, in both single- and mixed-species biofilm models.

**Results:**

Fourier transform infrared spectroscopy (FTIR) analysis revealed absorption bands consistent with a lipopeptide biosurfactant. F7 reduced surface tension to 22 mN/m (critical micelle concentration (CMC) 158 mg/L; emulsification index 63%), confirming strong surface activity. No planktonic minimum inhibitory concentration (MIC) or minimum bactericidal concentration (MBC)/minimum fungicidal concentration (MFC) was observed within 0.6–10 mg/mL. However, at 10 mg/mL, the minimum biofilm inhibitory concentration (MBIC) of F7 against single-species were 43.5% (*S. mutans*), 40.9% (*E. faecalis*), and 78.8% (*C. albicans*), and the minimum biofilm eradication concentration (MBEC) were 50.0%, 43.5%, and 34.8%, respectively. In mixed-species models at 10 mg/mL, MBIC was 23.4% (*S. mutans*-*C. albicans*), 44.7% (*E. faecalis*-*C. albicans*), and 27.6% (three-species), whereas MBEC was 74.0% (*S. mutans*-*C. albicans*), 42.8% (*E. faecalis*-*C. albicans*), and 42.5% (three-species). Resazurin assays demonstrated concordant metabolic suppression, and field emission scanning electron microscopy (FESEM) revealed membrane and matrix-associated structural alterations consistent with surface-active lipopeptide activity.

**Conclusion:**

Under the conditions tested, F7 functioned as an antibiofilm agent rather than a classical planktonic biocide. Notably, strong biofilm inhibition was observed against *C. albicans*, and the greatest biofilm reduction occurred in the mixed *S. mutans–C. albicans* consortium, accompanied by suppression of metabolic viability. These findings support a biofilm-modulating mechanism and suggest the potential of *B. clausii*-derived biosurfactants as non-biocidal adjuncts in oral biofilm management.

**Supplementary Information:**

The online version contains supplementary material available at https://doi.org/10.1186/s12866-026-05270-7.

## Background

Biosurfactants, synthesized by various microorganisms, are amphiphilic compounds containing both hydrophobic and hydrophilic regions. They offer several benefits over chemical surfactants, including lower toxicity, greater biodegradability, environmental friendliness, improved foaming ability, and effectiveness across a wide range of pH, temperature, and salinity conditions [1–3]. Due to their versatile applications, biosurfactants are often referred to as “sustainable,” “bio-based,” or “green” materials [4].

The production of biosurfactants relies heavily on microbial fermentation using bacterial, fungal, and yeast strains. Among the most recognized biosurfactants are lipopeptides produced by *Bacillus* species. Lipopeptides, in particular, have shown significant potential in inhibiting bacterial biofilms due to their stability and efficacy [5,6]. *Bacillus clausii*, isolated from a crude oil reservoir in Sumatra, Indonesia, produces the F7 biosurfactant, which exhibits exceptional stability across various pH, temperature, and salinity conditions, along with strong emulsifying properties [7]. The F7 biosurfactant reduces surface tension, disrupts soil and bio-corrosion bacteria, enhances smear layer removal in root canals, and improves antimicrobial penetration into biofilms [7, 8].

Despite growing interest in biosurfactants, few studies have investigated their antimicrobial and antibiofilm potential specifically against oral pathogens. The F7 biosurfactant, in particular, has not been evaluated for its efficacy against *S. mutans*, *E. faecalis*, and *C. albicans*, major contributors to oral biofilm formation. *E. faecalis*, a persistent biofilm-forming pathogen implicated in failed endodontic treatment; *C. albicans*, the predominant fungal contributor to mixed-species oral biofilms; and *S. mutans*, the principal cariogenic organism whose biofilm formation is driven by well-characterized virulence determinants including glucosyltransferases (gtfB, gtfC, gtfD) and the vicRK and liaSR two-component systems. These pathogens frequently form microbial biofilms that adhere to surfaces, protecting against environmental stress and antimicrobial agents [9, 10].

This study evaluates the antimicrobial and antibiofilm effects of F7 on planktonic and biofilm cells, including mixed-species biofilms, and examines treatment-induced ultrastructural changes. The findings provide foundational insights into F7’s potential as a biofilm-modulating agent in oral systems and guide future translational research.

## Methods

### Strains, growth media, and controls

*B. clausii*, isolated from a wellhead oil reservoir in Sumatra, Indonesia, was used as the biosurfactant producer [7]. The microbial strains tested included *S. mutans* (ATCC^®^ 25175™), *E. faecalis* (ATCC^®^ 24212™), and *C. albicans* (ATCC^®^ 14503™), cultured in brain heart infusion, tryptic soy, and sabouraud dextrose media (OXOID™), respectively. Chlorhexidine gluconate 0.2% (CHX), sodium hypochlorite 2.5% (NaOCl), and nystatin oral suspension 100,000 IU/mL (Tystatin™) were used as positive controls for *S. mutans*,* E. faecalis*, and *C. albicans*, respectively, with deionized water as the negative control.

### Preparation of the standard single-species microbial cell suspension

Colonies were harvested and suspended in appropriate broth: brain heart infusion (BHI) broth for *S. mutans*, tryptic soy broth (TSB) for *E. faecalis*, and sabouraud dextrose broth (SDB) for *C. albicans*. The turbidity of each suspension was then adjusted to the McFarland 0.5 standard equivalent to 10⁸ cells/mL [11–13].

### Preparation of the standard mixed-species microbial cell suspension

Mixed-species cultures were prepared by inoculating each test strain into its respective broth, then centrifuging at 10,000 × g for 10 min at 4 °C. The pellets were washed with phosphate-buffered saline (PBS), resuspended in BHI broth, and adjusted to a McFarland 0.5 standard (10⁸ cells/mL). The cultures were then combined in 1:1 volume ratios to create *S. mutans*–*C. albicans*, *E. faecalis*–*C. albicans*, and *S. mutans*–*E. faecalis*–*C. albicans* mixtures [12, 14, 15].

### Production and extraction of F7 biosurfactant

*B. clausii* was activated by inoculating 2–3 colony isolates into 50 mL of nutrient broth (NB) and incubating at 37 °C, 125 rpm for 24 h. A 10% (v/v) aliquot of this culture was transferred to fresh NB and incubated at 50 °C, 125 rpm for another 24 h. For adaptation, 10% (v/v) of the activated culture was inoculated into 150 mL of stone mineral salt solution extract yeast (SMSSe) with molasses and incubated at 50 °C, 125 rpm for 24 h. Subsequently, 10% (v/v) of the adapted culture was transferred into 1 L of SMSSe with molasses and incubated under the same conditions for 84 h. After production, the biosurfactant was extracted from the cell-free supernatant using a chloroform-methanol (2:1) mixture, and the organic phase was dried under acid airflow for 2–3 days to obtain a dry extract [7, 16].

### Biosurfactant stock solution and dilutions

The crude F7 biosurfactant was dissolved in deionized water (10 mg/mL), filtered through 0.2 μm polytetrafluoroethylene (PTFE) syringe filters, and stored at 4 °C. For experiments, concentrations of 0.6, 1.25, 2.5, 5, and 10 mg/mL were prepared.

### Characterization of F7 biosurfactant

Surface tension was measured using the Du-Nouy-ring method, and CMC was determined by plotting surface tension against F7 concentrations [2, 3]. Surface tension was measured in triplicate at 20–25 °C using a Dynamic 86 contact angle analyzer (KRUSS GmbH) with a 1.9 cm Du-Nouy-ring, calibrated with distilled water. The emulsification index was calculated after mixing crude oil with F7 biosurfactant (1:1), vortexing for 2 minutes, and measuring the emulsion height after 24 h. The emulsification index (%) was calculated as (emulsion layer height ÷ total liquid height) × 100 [2]. The FTIR (Nicolet 6700 FTIR Spectrometer, Thermo Fisher Scientific, Massachusetts, USA) analysis was conducted on potassium bromide (KBr) pellets containing F7, scanning the 4000–650 cm⁻¹ range [5].

### Growth profiles

Five milliliters of microbial cell suspension were added to 40 mL of liquid medium in conical flasks, along with 5 mL of sterile deionized water, for a total volume of 50 mL. The flasks were incubated at 37 °C in a shaker for 18 h, with optical density (OD) monitored hourly. Each test was performed in triplicate [17, 18].

### Biofilm profiles

Twenty milliliters of single and mixed-species suspensions were added to petri dishes with glass slides and incubated at 37 °C. The slides were weighed every 4 h for 48 h to measure biofilm wet weight, calculated as the difference between the biofilm-covered and bare slide. Each test was done in triplicate [12, 14, 15].

### Antimicrobial assay

A 100 µL microbial suspension was spread onto agar plates, with six 6 mm wells created using a sterile cork borer. Each well received 100 µL of F7 biosurfactant at varying concentrations (0.6, 1.25, 2.5, and 5 mg/mL) and controls. Inhibition zones were measured after 24-hour incubation at 37 °C. The MIC was determined by broth microdilution, in which serial dilutions of F7 biosurfactant and broth were prepared to achieve final concentrations of 0.6, 1.25, 2.5, and 5 mg/mL, and the microbial wash suspension was then added. Each test was performed in triplicate. The MIC was the lowest concentration at which no visible growth was observed. For MBC and MFC, 50 µL from the MIC wells were plated and incubated to determine the concentration at which no growth occurred [13, 18].

### Antiadhesive and antibiofilm assay

Antiadhesive activity was assessed by determining the MBIC, and antibiofilm activity was tested by evaluating the MBEC using crystal violet staining and a modified broth microdilution method. ^7^ For MBIC, 100 µL of single or mixed-species suspensions and 100 µL of biosurfactant at concentrations of 0.6, 1.25, 2.5, 5, and 10 mg/mL were incubated with controls at 37 °C. For MBEC, 200 µL of species suspension was incubated, washed with PBS, and treated with biosurfactant. The single and mixed biofilms were incubated according to their respective biofilm profiles. Each test was performed in triplicate. Following incubation, absorbance was measured at 600 nm using a Shimadzu UV-160 A spectrophotometer (Shimadzu, Japan), and inhibition percentages were calculated as follows:

$${\%}\;\text{Growth Inhibition}=[1-\text{(Ac / Ao)}]\times100$$

Ac was the absorbance of the well containing a biosurfactant concentration, while Ao signifies the absorbance of the negative control well [19].

### Cellular viability assay

Biofilm metabolic activity was quantified using a resazurin (alamarBlue) assay adapted from Brown et al. (2022) with minor modifications [20]. Single-species biofilms were cultivated in 96-well microtiter plates using BHI broth for *S. mutans*, TSB for *E. faecalis*, and SDB for *C. albicans*. Each well received 100 µL of microbial suspension and 100 µL of F7 biosurfactant (10,000 ppm); untreated controls received deionized water. Plates were incubated at 37 °C for 12 h (*S. mutans*), 16 h (*E. faecalis*), or 36 h (*C. albicans*). For the resazurin viability assay, a 0.001% resazurin solution (prepared from a 1% stock in PBS) was added (100 µL/well) and incubated for 1 h at 37 °C. Absorbance was measured at 570 and 600 nm, and cell viability (%) was calculated as:

$$\begin{array}{ll}\mathrm{Viability}\;({\%})=((\text{Asample -- Ablank})\mathrm{treated})\\/((\text{Asample -- Ablank})\mathrm{control})\times100\end{array}$$

A color shift from blue to pink indicates metabolic activity; lower percentage values denote stronger biofilm inhibition by the biosurfactant.

### Field Emission Scanning electron microscopy (FESEM)

Each microbial cell suspension was incubated until a mature biofilm formed, then mixed with F7 biosurfactant at 10 mg/mL in a 1:1 (v/v) ratio and incubated at 37 °C for 1 h. The suspensions were then fixed in 4% glutaraldehyde, washed with cacodylate buffer, and dehydrated using increasing ethanol concentrations (30% to 100%). The samples were further fixed in pure acetone, subjected to critical point drying (CPD), and analyzed using a FESEM FEI Quanta FEG 650 S.

### Statistical analysis

Analyses were performed in SPSS v22.0 (IBM SPSS Statistics). Data were screened for normality (Shapiro–Wilk test) and, when applicable, for homogeneity of variance (Levene’s test). Normally distributed data with equal variances were compared using one-way ANOVA with Tukey’s post-hoc test; results are reported as F(df1, df2), p. When assumptions were not met, we used the Kruskal–Wallis H test followed by Dunn’s post-hoc tests with Bonferroni adjustment; results are reported as H(df), p (two-tailed). Unless stated otherwise, values are mean ± standard deviation (SD), and α = 0.05. Each condition was measured in three technical wells in a single experimental run (*n* = 3 technical; *n* = 1 biological). As independent biological replicates were not performed, statistical analyses primarily indicate technical consistency.

## Results

### Production and characterization of F7 biosurfactant

**Fig. 1:**
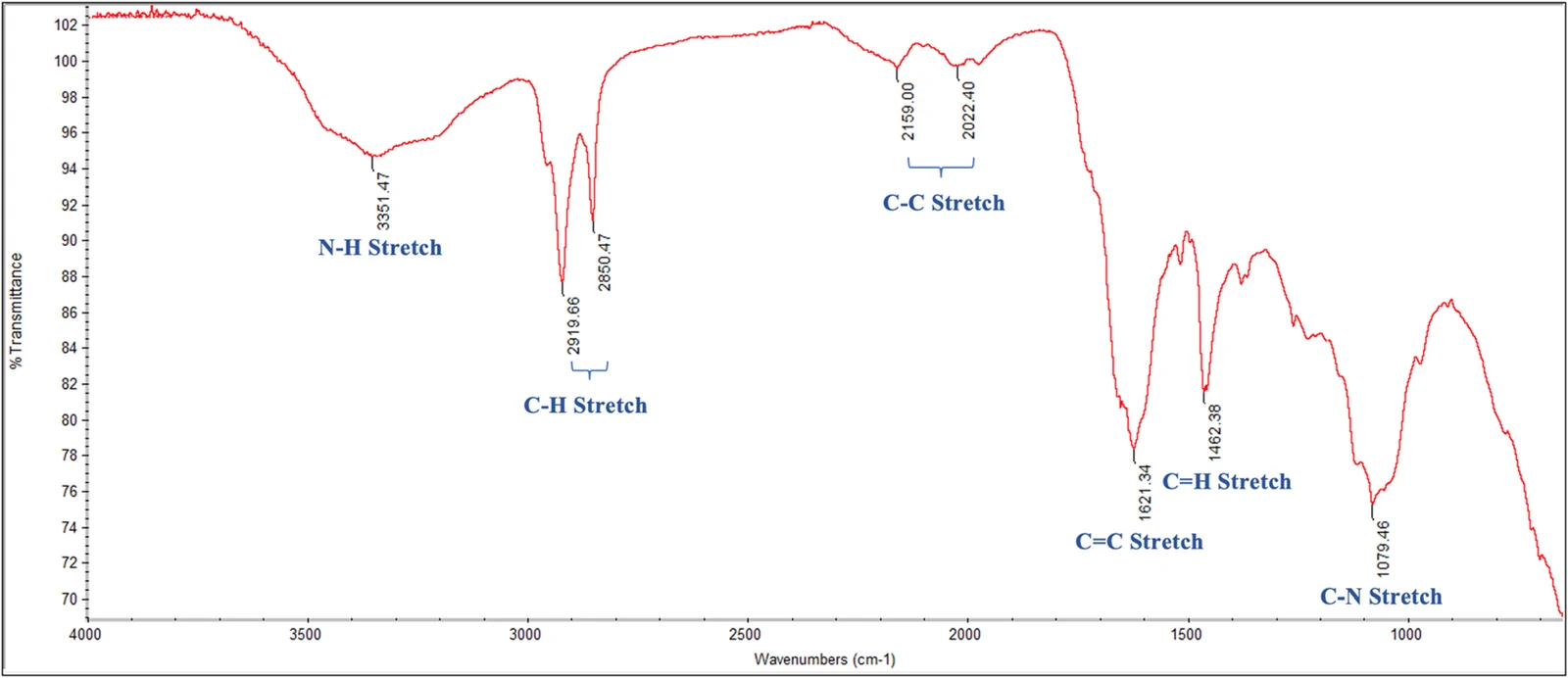
FTIR spectrum of F7 biosurfactant

A total of 1.29 g of dry F7 biosurfactant extract was produced, yielding a stock solution at 129 mg/mL. For testing purposes, the F7 biosurfactant was diluted to a final concentration of 10 mg/mL. The results demonstrated that the F7 biosurfactant reduced the surface tension by 22 mN/m, with a CMC of 157.5 mg/L. The emulsification index of the F7 biosurfactant was calculated to be 63%. The FTIR spectrum exhibited several distinct absorption peaks, indicating the presence of various functional groups within the material, as shown in Fig. 1.

The figure shows that the band observed at 3351.47 cm⁻¹ corresponds to a carbon-containing compound with an amino group (N-H stretching), indicating the lipopeptide nature of the molecule [21]. Peaks at 2919.66 cm⁻¹ and 2850.47 cm⁻¹, as well as at 1462.38 cm⁻¹, are assigned to asymmetric and symmetric C–H stretching of aliphatic CH₂ and CH₃ groups, reflecting the hydrocarbon chains of the lipid moiety. The band at 1621.34 cm⁻¹ is attributed to C = O stretching (amide I), further supporting the presence of peptide linkages [21, 22]. Additionally, absorption between 2159.00 cm⁻¹ and 2022.40 cm⁻¹ may involve other C–C and C = C stretching vibrations in the backbone [22]. The peak observed at 1079.46 cm⁻¹ is consistent with C–N stretching, indicating peptide groups within the biosurfactant molecule [21, 22].

### Antimicrobial properties of F7 biosurfactant

Across 0.6–10 mg/mL, F7 produced no inhibition zones in agar diffusion. In broth microdilution, MICs were not reached for *S. mutans* (12 h), *E. faecalis* (12 h), or *C. albicans* (18 h), with persistent turbidity across all concentrations (MIC > 10 mg/mL). Plate counts confirmed the absence of a bactericidal/fungicidal threshold (MBC/MFC > 10 mg/mL). Growth profiles–time plots are provided in Supplementary Figure S1.

### Antiadhesive and antibiofilm properties of F7 biosurfactant

The incubation times based on biofilm growth curves were set as follows: 12 h for *S. mutans*, 16 h for *E. faecalis*, 36 h for *C. albicans*, 36 h for the *S. mutans and C. albicans* mixed biofilm, and 40 h for both the *E. faecalis* and *C. albicans* mixed biofilm and the three-species mixed biofilm, yielding the highest biofilm wet weight. Supplementary data provide detailed biofilm profiles (Supplementary Figure S2 and Figure S3).

**Fig. 2:**
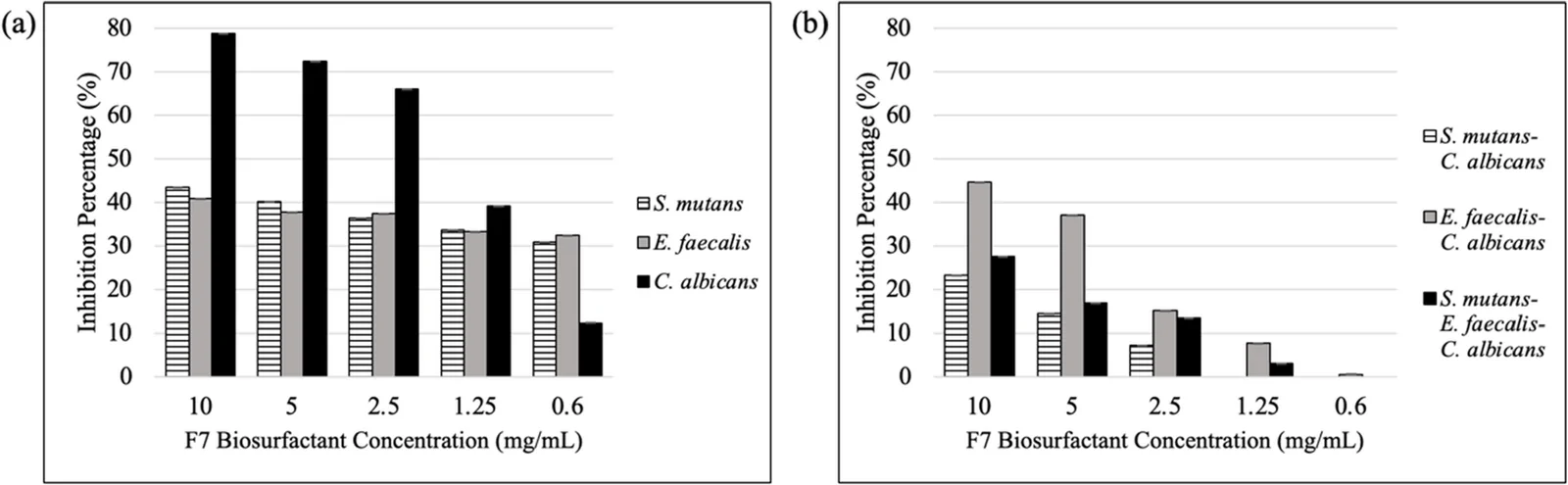
Inhibition of biofilm formation (MBIC) by F7 biosurfactant. (**a**) Single-species and (**b**) mixed-species models across 0.6–10 mg/mL. Data are presented as mean ± SD of technical triplicates (*n* = 3). Statistical analysis was performed using one-way ANOVA for single-species data and the Kruskal–Wallis H test for mixed-species data, with *p*-values > 0.05. Error bars may be smaller than the symbol size

In single-species assays, F7 reduced biofilm formation in a dose-dependent manner for all organisms. Detectable inhibition was observed at 0.6 mg/mL, and ≥ 50% inhibition of *C. albicans* was achieved at 2.5 mg/mL. At 10 mg/mL, inhibition was 43.5% ± 0.001 for *S. mutans*, 40.9% ± 0.002 for *E. faecalis*, and 78.8% ± 0.006 for *C. albicans* (Fig. 2a). Relative to the positive controls, responses were comparable for *S. mutans* (CHX, 43.5% ± 0.002), lower for *E. faecalis* (2.5% NaOCl, 83.6% ± 0.001), and moderately lower for *C. albicans* (nystatin 100,000 IU/mL, 83.5% ± 0.009). In mixed-species consortia, inhibition was dose-dependent and generally lower than in single-species models. At 10 mg/mL, inhibition was 23.4% ± 0.006 for *S. mutans-C. albicans*, 44.7% ± 0.005 for *E. faecalis-C. albicans*, and 27.6% ± 0.007 for the three-species consortium (Fig. 2b). At the same endpoint, the positive controls yielded 41.3% ± 0.005, 73.9% ± 0.009, and 81.6% ± 0.001, respectively. All values are reported as mean ± SD of technical triplicates.

**Fig. 3:**
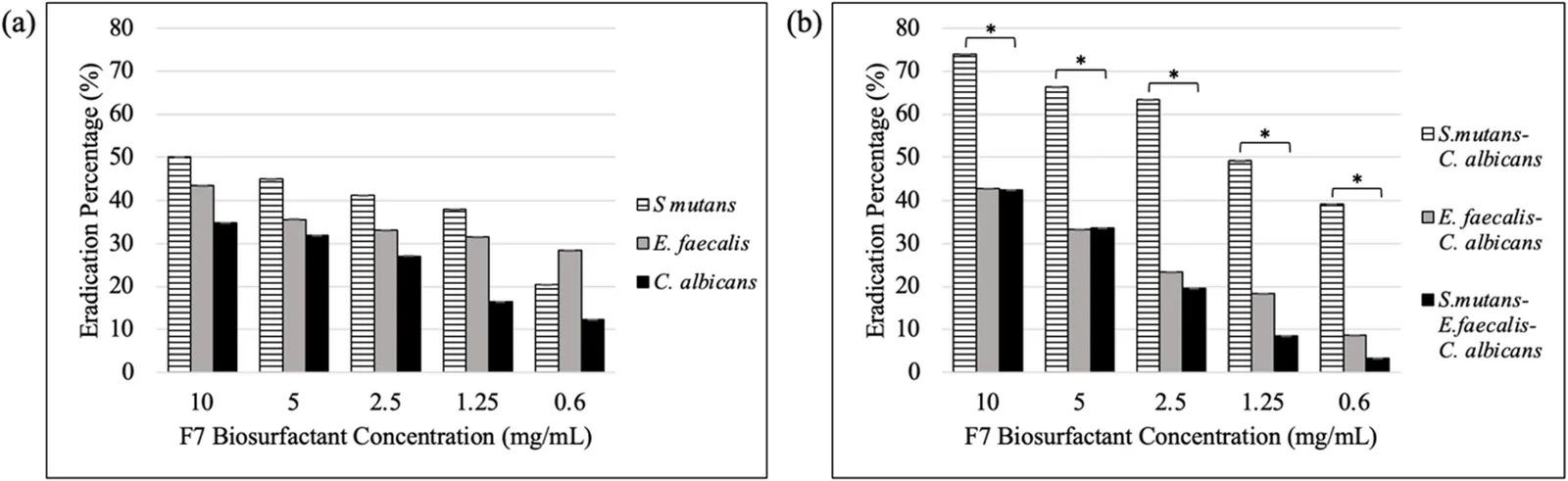
MBEC by F7 biosurfactant. (**a**) Single-species and (**b**) mixed-species models across 0.6–10 mg/mL. Data are presented as mean ± SD of technical triplicates (*n* = 3). Statistical analysis was performed using the Kruskal–Wallis H test for single-species and mixed-species data. Bars show mean ± SD of technical triplicates (*n* = 3). The asterisk (*) denotes a statistically significant difference between the *S. mutans*-*C. albicans* consortium and the three-species consortium (*P* < 0.05). Error bars may be smaller than the symbol size

The MBEC of F7 biosurfactant exhibited a concentration-dependent effect in the pre-formed biofilms evaluation (Fig. 3). In single-species assays, treatment at 10 mg/mL markedly reduced pre-formed biofilms by 50.0% ± 0.009 for *S. mutans*, 43.5% ± 0.002 for *E. faecalis*, and 34.8% ± 0.006 for *C. albicans* (Fig. 3a). In mixed-species consortia, biofilm reduction at 10 mg/mL reached 74% ± 0.009 for *S. mutans*-*C. albicans*, 42.8% ± 0.009 for *E. faecalis*-*C. albicans*, and 42.5% ± 0.006 for the three-species model (Fig. 3b). All values are reported as mean ± SD of technical triplicates. Among single species, *S. mutans* showed the greatest reduction, while the *S. mutans*-*C. albicans* consortium exhibited the highest response overall (74%). A Kruskal–Wallis H test indicated significant differences among groups (*p* < 0.05), and Dunn’s post-hoc with Bonferroni adjustment showed greater eradication for *S. mutans-C. albicans* versus the three-species consortium at 10 mg/mL (adjusted *p* < 0.05).

### Cellular viability assay

Based on the viability assay results shown in the figure, the F7 biosurfactant exhibited a concentration-dependent reduction in the viability of *S. mutans*, *E. faecalis*, and *C. albicans* (*n* = 3 technical wells/condition). At 10 mg/mL, residual viability (vehicle-normalized) was 26.16% for *S. mutans*, 24.55% for *E. faecalis*, and 27.13% for *C. albicans*, corresponding to 73.84%, 75.45%, and 72.87% reductions compared to the control, respectively. As the concentration decreased, a progressive decline in viability was observed across all test organisms. Overall, the F7 biosurfactant significantly reduced cellular viability across all tested microorganisms. Observed reductions in cell viability indicate antimicrobial activity, rather than direct confirmation of biofilm-specific effects. *S. mutans* exhibited the greatest susceptibility, while *C. albicans* was the least affected, reflecting the viability-reduction effects on the tested microbial cells.

**Fig. 4:**
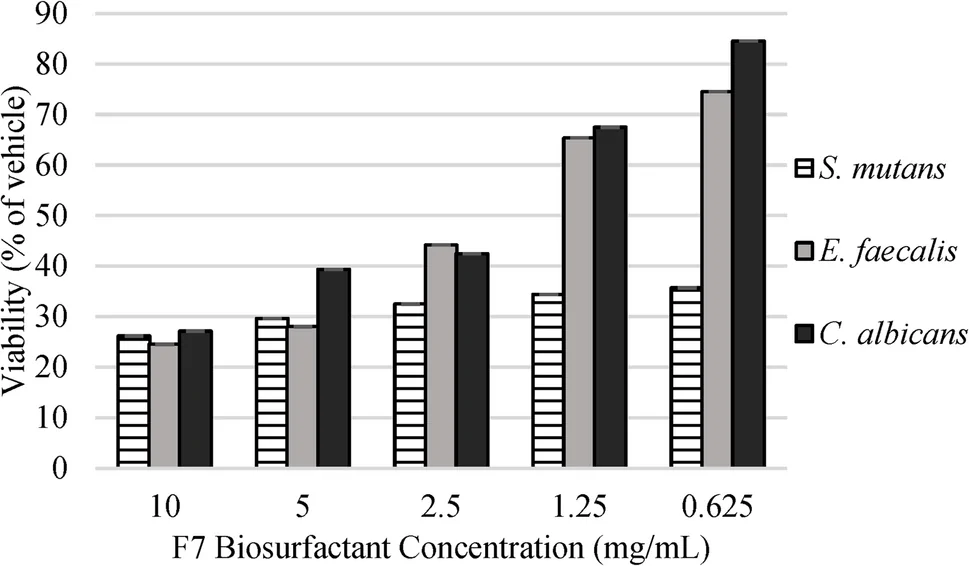
Metabolic viability activity in a pre-formed biofilm after treatment by F7 biosurfactant using the Resazurin assay. Data are presented as mean ± SD of technical triplicates (*n* = 3). Statistical analysis was performed using the Kruskal–Wallis H test for single-species and mixed-species data. Bars show mean ± SD of technical triplicates (*n* = 3); error bars may be smaller than symbol size

Below is the summarized table to facilitate comparison across biofilm models and experimental endpoints at 10 mg/mL.

**Table 1:** Summary of antibiofilm and viability outcomes of F7 biosurfactant at 10 mg/mL

Biofilm Model	Biofilm Type	Biofilm Inhibition (%)		Biofilm Reduction (%)		Viability reduction (%)	
		F7	Positive controls	F7	Positive controls	F7	Positive controls
*S. mutans*	Single-species	43.5	43.5	50.0	36.4	26.16	29.62
*E. faecalis*	Single-species	40.9	83.6	43.5	52.7	24.55	14.34
*C. albicans*	Single-species	78.8	83.5	34.8	37.9	27.13	23.58
*S. mutans-C. albicans*	Dual-species	23.4	41.3	74.0	59.8	-	-
*E. faecalis-C. albicans*	Dual-species	44.7	73.9	42.8	62.2	-	-
Three-species consortium	Mixed-species	27.6	81.6	42.5	44.7	-	-

Values represent percentage of MBIC, percentage of MBEC, and reduction in cellular viability relative to untreated controls at 10 mg/mL of F7 biosurfactant, as determined by crystal violet and resazurin assays. Positive-control values are shown for comparison

### Ultrastructural cell morphological analysis using FESEM

**Fig. 5:**
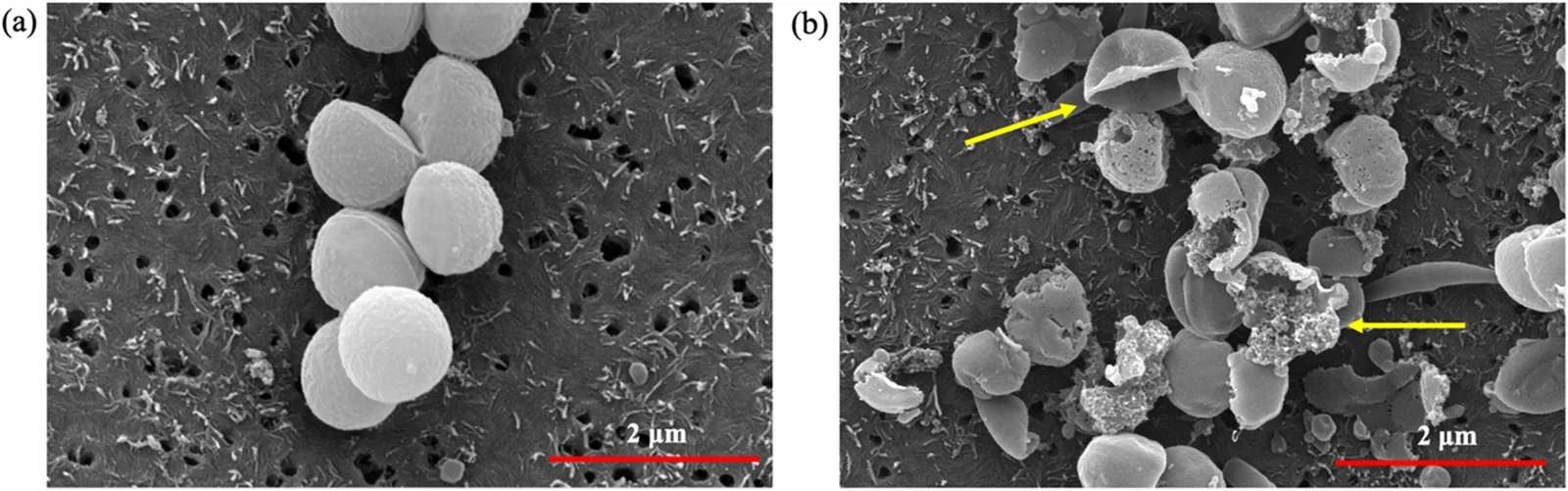
FESEM micrographs of untreated and F7-treated *S. mutans* cells. **a** The untreated control showed intact cell morphology and smooth cell surfaces. **b** Cells treated with 10 mg/mL F7 biosurfactant showed disrupted cell membranes and surface deformation (yellow arrow). Images were acquired at 60,000 x magnification, with scale bars shown in each panel

**Fig. 6:**
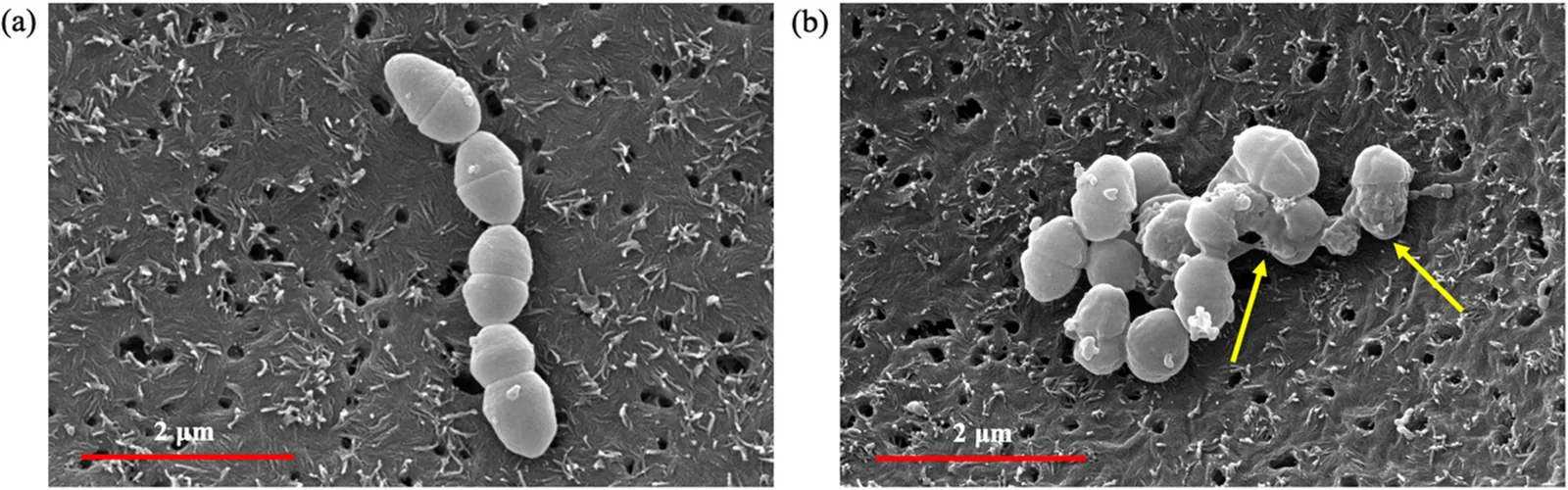
FESEM micrographs of untreated and F7-treated *E. faecalis* cells. **a** The untreated control exhibited typical coccal morphology and intact cell walls. **b** Treatment with 10 mg/mL F7 biosurfactant caused membrane damage and cell-surface irregularities (yellow arrow). Images were acquired at 60,000 x magnification, with scale bars shown in each panel

**Fig. 7:**
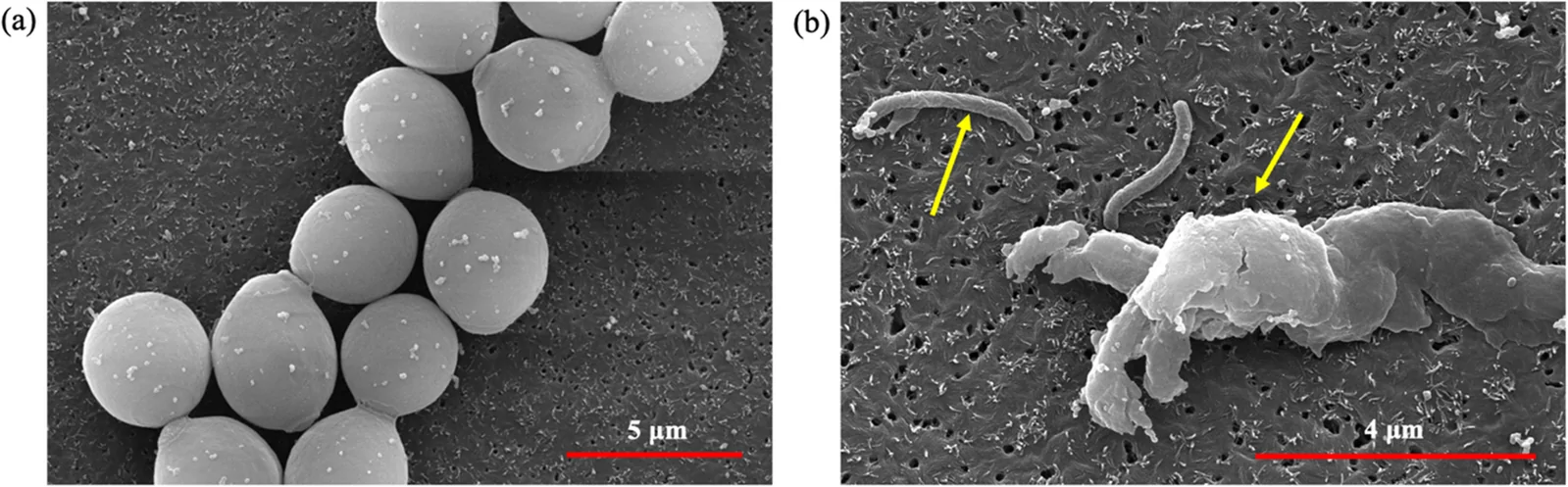
FESEM micrographs of untreated and F7-treated *C. albicans*. **a** Untreated control displaying intact yeast cells (20,000 x magnification). **b** Cells treated with 10 mg/mL F7 biosurfactant exhibited morphological alterations, including hyphal detachment and surface disruption (yellow arrow) (40,000x magnification). All images include scale bars, and magnifications are indicated in the respective panels

Untreated *S. mutans* cells were spherical, with smooth surfaces and intact membranes. In contrast, cells treated with 10 mg/mL of F7 biosurfactant showed membrane damage, puncturing, and cytoplasmic leakage (Fig. 5). Similarly, untreated *E. faecalis* cells appeared smooth and intact, forming chains, while treated cells became misshapen, lost their smooth surfaces, and no longer formed chains (Fig. 6). Untreated *C. albicans* cells had regular spherical shapes with intact membranes, but after treatment, FESEM images revealed disrupted membranes, cell lysis, and detached hyphae (Fig. 7).

## Discussion

The discovery and production of novel biosurfactants are crucial for addressing economic challenges in biosurfactant production, given the limited availability of commercially produced options such as surfactin, sophorolipids, and rhamnolipids [23, 24]. The production of biosurfactants is influenced by carbon and nitrogen sources, pH, temperature, carbon-to-nitrogen (C/N) ratio, agitation, and oxygen availability [3, 25, 26]. In this study, we successfully extracted a crude F7 biosurfactant from *B. clausii* using SMSSe and molasses as nutrient sources, achieving a high yield [27]. The extraction employed the chloroform-methanol method and drying under acidic airflow, aligning with methods shown to be effective for lipopeptide extraction [28].

Biosurfactants are characterized by their ability to reduce surface and interfacial tension, thereby aiding the formation of stable emulsions [3]. In this study, the F7 biosurfactant reduced surface tension by 22 mN/m, meeting the effectiveness criterion of a reduction greater than 20 mN/m [29]. Its CMC was 157.5 mg/L, within the typical biosurfactant range of 1–200 mg/L, allowing the formation of stable nanostructures [16]. With an emulsification index of 63%, F7 effectively stabilizes emulsions [30]. FTIR analysis is indicative of functional groups rather than definitive molecular structures. Its lipopeptide nature aligned with previous findings on biosurfactants containing hydrophilic peptide bonds and hydrophobic aliphatic chains, similar to those reported in other *B. clausii* strains [16, 31, 32].

The growth profile of each microorganism was evaluated to determine the optimal incubation time, focusing on the stationary phase where growth stabilizes [33]. We observed no planktonic MIC/MBC/MFC within 0.6–10 mg/mL, yet F7 produced a dose-dependent MBIC and MBEC. MBIC was defined as the lowest concentration of F7 biosurfactant that inhibited biofilm formation, while MBEC was the lowest concentration that markedly reduced a pre-formed biofilm [7]. Because biofilm cells are phenotypically distinct from planktonic cells, compounds may fail to achieve classical MIC/MBC thresholds while still disrupting biofilm architecture, which is why MIC/MBC and MBIC/MBEC are not directly comparable [14, 24]. This dissociation suggests a non-classical antibiofilm profile in which surface modification, interfacial disruption, and matrix destabilization occur independently of planktonic growth inhibition. Such behaviour is increasingly recognized among biosurfactants and distinguishes them mechanistically from conventional biocides. Ultrastructural alterations observed by FESEM further support membrane and matrix-associated effects, although definitive mechanistic pathways remain to be elucidated.

The comparatively stronger inhibition observed against *C. albicans* and the pronounced eradication of the *S. mutans–C. albicans* dual-species biofilm may reflect the amphiphilic architecture of lipopeptide biosurfactants. Their hydrophobic fatty acid chains and hydrophilic peptide domains enable interaction with lipid bilayers, potentially facilitating perturbation of ergosterol-rich fungal membranes. Disruption of fungal structural components may, in turn, weaken interspecies biofilm cohesion, thereby contributing to the observed reduction in the dual-species consortium [34–36].

The predominant activity of F7 against the budding yeast form of *C. albicans*, together with hyphal detachment rather than direct hyphal lysis observed by FESEM (Fig. 7), is consistent with a surface-active, antiadhesive mechanism rather than direct fungicidal action. Mature *C. albicans* biofilms develop in a layered architecture, with a basal yeast monolayer that anchors a superficial network of pseudohyphae and hyphae embedded in a β-1,3-glucan-rich extracellular matrix [37, 38]. Disruption of the basal yeast adhesion layer by the surface-active activity of F7 may therefore destabilize hyphal anchoring, producing the hyphal detachment observed by FESEM without direct hyphal killing. This interpretation is consistent with the absence of MIC/MFC within the tested range and reinforces the antiadhesive rather than biocidal profile of F7 demonstrated in this study.

Although inhibition was consistent across multiple species, some effects were moderate, and MIC/MBC values were not achieved within the tested range. Similar activity has been reported for crude biosurfactants, including those from *Lactobacillus plantarum*, which show negligible planktonic killing at concentrations up to 50 mg/mL against endodontic pathogens [39]. While the precise mechanism requires confirmation, biosurfactants can lower interfacial tension, compete for surface sites, and insert into lipid bilayers, increasing membrane permeability and perturbing electrochemical homeostasis (proton-motive force/ion flux), with downstream effects on transport and energy metabolism [24, 40]. In the oral context, recent evidence synthesizes their antibiofilm potential and delivery considerations [41].

In our biofilm assays, F7 produced clear dose-dependent reductions in formation and partial eradication across organisms. Higher F7 concentrations enhanced inhibition, consistent with reports that biosurfactants from *Lactobacillus* strains reduce *S. mutans/S. oralis* biofilms on titanium, and that rhamnolipids increase bacterial membrane permeabilization [42, 43].

FESEM revealed morphological changes across all strains following F7 treatment. These ultrastructural changes could reflect interactions of biosurfactant alkyl chains with cell membranes, which may influence membrane structure or stability. The lack of planktonic antimicrobial thresholds (MIC/MBC/MFC), despite pronounced morphological changes, suggests altered permeability and interfacial effects rather than rapid cell death under the tested conditions [44–46].

Compared with standard oral antimicrobial agents such as chlorhexidine, sodium hypochlorite, and nystatin, which exhibit strong biocidal activity against planktonic bacteria and established biofilms, F7 primarily displayed surface-active and antiadhesive effects rather than direct antimicrobial potency. While CHX, NaOCl, and nystatin rapidly reduce viable cell counts in biofilms, biosurfactants such as surfactin inhibit biofilm formation and reduce biofilm biomass by interfering with microbial adhesion and extracellular matrix production [47, 48] The biofilm-modulating effects observed for F7 are consistent with reports on other surfactins, which have been shown to reduce biofilm formation and disrupt pre-formed biofilms in various bacterial species. Such activities are attributed to their surface-active properties and ability to interfere with microbial adhesion and matrix stability, supporting the potential of F7 as a complementary antibiofilm agent [48, 49].

This study has several important limitations. The biosurfactant was evaluated as a crude extract, without purification or compositional profiling, which limited the identification of specific active constituents. Furthermore, the simplified in vitro models employed do not fully replicate the complexity of the oral environment, where saliva, host-derived factors, multispecies interactions, and mechanical forces influence biofilm behaviour and treatment response. Accordingly, these findings should be interpreted as exploratory characterization rather than definitive translational validation. Future investigations should include purification and molecular characterization of F7, independent biological replicates, extracellular matrix analysis, cytocompatibility assessment, and evaluation in saliva-conditioned, clinically relevant, multispecies biofilm models. Nevertheless, this study represents the first evaluation of a *B. clausii*–derived biosurfactant in oral multispecies biofilm models. It supports the emerging concept of non-biocidal, biofilm-modulating strategies in oral microbiology.

## Conclusion

Under the tested conditions, the crude lipopeptide biosurfactant F7 demonstrated antibiofilm activity consistent with a surface-active, antiadhesive mechanism rather than a primary planktonic biocidal effect. Activity was model- and endpoint-dependent, with greater inhibition of biofilm formation in single-species systems (notably *C. albicans*) and greater disruption of established biofilm in the mixed *S. mutans–C. albicans* model. Surface-tension reduction (low CMC) and FESEM-observed structural alterations support membrane and matrix-associated effects, although the precise mechanisms remain to be fully elucidated.

Collectively, these findings suggest that F7 may have adjunctive potential in strategies aimed at modulating oral biofilms, particularly fungal-associated and mixed-species plaque biofilms. Further purification and validation in clinically relevant models are required before translational application.

## Supplementary Information

Supplementary Material 1.

Supplementary Material 2.

Supplementary Material 3.

## Data Availability

All data generated or analyzed during this study are included in this published article [and its supplementary information files].

## Abbreviations

ANOVA: Analysis of variance
ATCC: American Type Culture Collection
BHI: Brain Heart Infusion
C. albicans: Candida albicans
CHX: Chlorhexidine
CMC: Critical micelle concentration
E. faecalis: Enterococcus faecalis
FESEM: Field-emission scanning electron microscopy
FTIR: Fourier transform infrared spectroscopy
KBr: Potassium bromide
MBEC: Minimum biofilm eradication concentration
MBIC: Minimum biofilm inhibitory concentration
MBC/MFC: Minimum bactericidal/fungicidal concentration
MIC: Minimum inhibitory concentration
NaOCl: Sodium hypochlorite
NB: Nutrient broth
OD: Optical density
PBS: Phosphate-buffered saline
PTFE: Polytetrafluoroethylene
Rpm: Revolutions per minute
S. mutans: Streptococcus mutans
SDB: Sabouraud dextrose broth
SD: Standard deviation
SMSSe: Stone mineral salt solution extract yeast
TSB: Tryptic soy broth
